# H7 Hemagglutinin nanoparticles retain immunogenicity after >3 months of 25°C storage

**DOI:** 10.1371/journal.pone.0202300

**Published:** 2018-08-09

**Authors:** Timothy Z. Chang, Lei Deng, Bao-Zhong Wang, Julie A. Champion

**Affiliations:** 1 School of Chemical & Biomolecular Engineering, Georgia Institute of Technology, Atlanta, GA, United States of America; 2 Institute for Biomedical Sciences, Georgia State University, Atlanta, GA, United States of America; University of Georgia, UNITED STATES

## Abstract

Vaccine distribution infrastructure remains inadequate in many parts of the world, and it is estimated that up to 40–50% of all vaccine doses are wasted in certain countries. Vaccines that can maintain viability outside of the cold chain would decrease vaccine wastage and increase immunization rates in regions of the world with underdeveloped vaccine distribution infrastructure. We examined the potential of crosslinked protein nanoparticles, made from trimerized influenza hemagglutinin (3HA), to maintain immunogenicity after cold-chain-independent storage. We found that the nanoparticles could be stored for 112 days at room temperature without any loss in hemagglutinating activity or immunogenicity, and that nanoparticles could be stored at 37°C for 2 weeks without any loss in hemagglutinating activity. As vaccine development moves towards the use of recombinant subunit antigens, our results demonstrate the potential of crosslinked antigen nanoparticles as an immunogenic vehicle for bringing effective vaccines to underdeveloped regions outside of the cold chain.

## Introduction

Despite advances in vaccine development, vaccine distribution infrastructure remains inadequate in many parts of the world, and it is estimated that up to 40–50% of all vaccine doses are wasted in certain countries[1]. Crucial to current vaccine transport is the idea of the cold-chain–a series of refrigerated enclosures with tight temperature control that allows for stable transport of vaccine from manufacturer to patient. Strict control of temperature is important for whole pathogen vaccines, as these are particularly prone to stability losses[2]. In addition, pathogens with lipid membranes, such as bacteria and certain viruses including influenza, are especially prone to osmotic stress, and changing salt concentrations due to temperature-driven solvent evaporation can lead to pathogen shrinkage and destruction[2, 3]. The development of vaccines that can maintain viability outside of the cold chain would decrease vaccine wastage and increase immunization rates in regions of the world with underdeveloped vaccine distribution infrastructure.

Although recombinant, subunit protein vaccines have been proposed as a more stable alternative to whole pathogen vaccines, issues with low immunogenicity and appropriate adjuvant choice have slowed their development as a viable option[2]. Protein nanoparticles, made entirely of crosslinked protein antigens, are a means of delivering antigen and adjuvant in the same delivery vehicle, and are thus an excellent candidate for testing cold-chain-independent vaccine stability[4–6].

Monomeric influenza hemagglutinin is a 63 kDa protein responsible for mediating viral entry into host cells[7]. We have shown previously that protein nanoparticles made from trimerized, H7 hemagglutinin (3HA) were able to protectively immunize mice against a 10xLD_50_ H7 influenza challenge[6]. Given the immunogenicity of these nanoparticles, as well as the *in vitro* hemagglutination assay that can provide a simple measurement of protein conformation in nanoparticles, we have examined the viability of cold-chain-independent storage of 3HA nanoparticles. We sought to assess whether storing nanoparticles at room temperature (~25°C) or 37°C for several months resulted in a loss of hemagglutinating capability or immunogenic potential. We found that nanoparticles stored at room temperature retained both hemagglutinating activity and immunogenicity, while nanoparticles stored at 37°C retained hemagglutinating activity for 2 weeks.

## Materials and methods

### 2.1 Nanoparticle synthesis and characterization

Trimerized H7 hemagglutinin (3HA) protein was produced and purified from Sf9 cell culture, and nanoparticles were synthesized and characterized exactly as described previously[6]. Briefly, 400 μL ethanol was added to 100 μL of a 1.6 mg/mL 3HA solution at a rate of 1 mL/min under constant stirring at 600 rpm. The particles were collected by centrifugation, and resuspended in sterile phosphate-buffered saline (PBS) with sonication. 800 μg soluble 3HA protein was added at a final concentration of 1.6 mg/mL to 480 μg desolvated 3HA nanoparticles and an amine crosslinking reaction was performed using 3 mM 3,3´-Dithiobis[sulfosuccinimidylpropionate (DTSSP, Thermo Scientific, Waltham, MA) for 12 hours while stirring to coat the nanoparticles. Coated nanoparticles were collected by centrifugation, and protein concentration was measured by a bicinchoninic acid (BCA) assay according to the manufacturer’s instructions (Thermo Scientific) to estimate the total protein content in nanoparticles. Dynamic light scattering (DLS) was performed in PBS with a Malvern Zetasizer Nano ZS (Malvern Instruments, Westborough, MA) to assess nanoparticle size distributions.

Hemagglutinating capability of 3HA nanoparticles was tested as previously described[6]. Briefly, 5 μg of 3HA protein or 3HA protein nanoparticles in 100 μL of PBS was serially diluted by half across 11 wells of a 96-well plate. 50 μL of a 0.5% turkey RBC suspension in saline was then added to each well, and incubated at room temperature for 1 hour. The hemagglutination titer was read as the last well in the serial dilution that did not form a red button of settled RBCs.

### 2.2 Extended storage

Extended storage of 3HA nanoparticles was performed in PBS at room temperature (25°C) or 37°C. Nanoparticles and soluble protein were diluted to 200 μg/mL, the concentration for vaccination, in 100 μL aliquots in 2 mL centrifuge tubes. The tubes were sealed with parafilm and incubated for up to 1 month at 37°C, and up to 112 days at room temperature. At each time point, one 100 μL aliquot was used to measure hemagglutination activity of the nanoparticles, while two 300 μL aliquots were stored at room temperature for *in vivo* immunizations.

### 2.3 Immunization and sample collection

All animal work was done in accordance with the IACUC guidelines of Georgia State University, which specifically approved this study under IACUC approval number A16024. Female, 6 week old Balb/c mice (Charles River, Wilmington, MA) were intra-muscularly immunized with 10 μg aged 3HA nanoparticles, 20 μg aged 3HA nanoparticles, 10 μg freshly prepared 3HA nanoparticles, 10 μg soluble 3HA protein, or PBS as a control. Five mice per group were immunized twice i.m. at a 3-week interval. To compare antibody responses, sera were collected 2 weeks after each immunization by submandibular venipuncture. No anesthesia or analgesia was used in this study. All animals were euthanized by CO_2_ asphyxiation according to the American Veterinary Medical Association Guidelines for the Euthanasia of Animals.

### 2.4 Serum IgG titer

Serum IgG titer was assessed as previously described [5, 6]. Briefly, ELISA plates were coated with 1 μg/mL 3HA protein in PBS and incubated overnight at room temperature. Plates were blocked with 1% bovine serum albumin (BSA) in PBS for 1 hour. Mouse serum samples were initially diluted 1:100 in 1% BSA, and serial half-dilutions were made in 1% BSA across the 96-well ELISA plate. Serum sample dilutions were incubated for 1 hour, and 1 μg/mL HRP-conjugated goat-anti-mouse IgG (Life Technologies, Grand Island, NY) in 1% BSA was used as a detection antibody. Chromogenic quantification was performed by the oxidation of tetramethylbenzidine by hydrogen peroxide (R&D Systems, Minneapolis, MN) according to the manufacturer’s instructions. Two times the absorbance of naïve group’s serum samples was considered the cutoff for measuring the endpoint titer.

### 2.5 Hemagglutination inhibition assessment

Serum hemagglutination inhibition (HAI) activity was assessed according to a protocol adapted from the World Health Organization[8]. 10 μL mouse serum was incubated with 30 μL of receptor-destroying enzyme (RDE) (Denka Seiken Co, Tokyo, Japan) at 37°C overnight, followed by 56°C incubation for 30 minutes to inactivate non-specific agglutinating proteins. Inactivated serum was diluted with 60 μL room temperature PBS, and centrifuged at 500xg for 8 minutes to collect treated serum samples. Eight, 25 μL serial half-dilutions of 1:10 treated sera were mixed in a round-bottom 96-well plate with 25 μL of 2 μg/mL 3HA protein and incubated at room temperature for 30 minutes. This concentration of 3HA corresponded to 8 Hemagglutination Units (HAU) in 50 μL of PBS, as prescribed by the WHO protocol [8]. To this mixture, 50 μL of a 0.5% turkey RBC suspension was added, and the wells were incubated for 1 hour to develop. HAI titer was read as the inverse dilution of the last well able to prevent hemagglutination, or in which a red button of settled RBCs was formed.

### 2.6 Statistics

IgG titers were assessed by the Kruskal-Wallis one-way analysis of variance (ANOVA) for non-parametric samples. Hemagglutination inhibition titers were analysed by comparing the number of wells neutralized to the standard HAI titer of 40 (2 wells) established by the US Food and Drug Administration (FDA) as protective, using a one-sample t test. P values less than 0.05 were considered significant.

## Results

**Fig 1:**
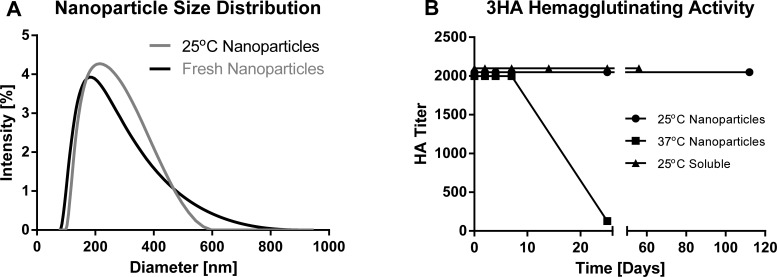
Physical characterization of 3HA nanoparticles. (A)Sizes of fresh 3HA nanoparticles (black) and 3HA nanoparticles stored at 25⁰C for 112 days (gray) were measured by dynamic light scattering (DLS). Each curve is representative of 3 independent DLS measurements taken per sample. (B)Hemagglutinating activity of 3HA nanoparticles or soluble 3HA protein over the course of approximately 3 months. Each data point represents the hemagglutinating activity of 1 sample tested.

H7 hemagglutinin nanoparticles were synthesized and characterized as described previously[6] (Fig 1A). The resulting nanoparticle size distribution was similar to that of previously synthesized batches of nanoparticles[6]. A pilot hemagglutination study of three replicates showed identical hemagglutinating titers (S1 Fig), and one replicate per timepoint was used for further hemagglutination assays to conserve materials. Nanoparticles stored in PBS at room temperature did not lose agglutinating activity or appreciably change in size after 112 days, while nanoparticles stored at 37°C retained full hemagglutinating activity for 2 weeks, and lost activity at 1 month (Fig 1A and 1B). Soluble 3HA in PBS showed similar stability to nanoparticles when stored at room temperature; no hemagglutinating activity losses were observed after 56 days.

**Fig 2:**
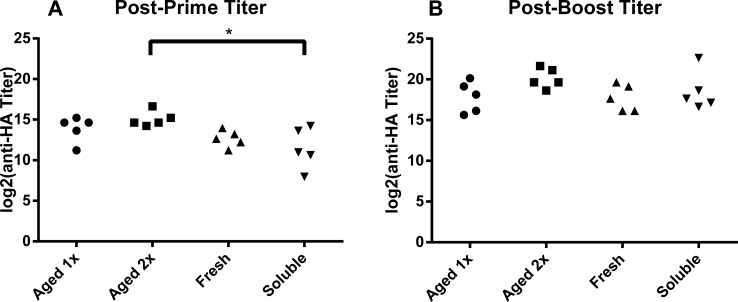
No significant difference in immunogenicity between warm-aged and freshly prepared 3HA nanoparticles. Serum IgG titers from mice 2 weeks after a priming immunization (A) or a boost immunization (B) of 10 μg room temperature-stored 3HA nanoparticles (Aged 1x), 20 μg room temperature-stored 3HA nanoparticles (Aged 2x), 10 μg freshly prepared 3HA nanoparticles (fresh) or 10 μg soluble 3HA (soluble). Each point is the IgG titer of one mouse. (* = p<0.05 by one-way ANOVA).

To compensate for any potential loss in hemagglutinating activity, we immunized mice with a single dose (10 μg) or double dose (20 μg) of nanoparticles stored for 112 days in PBS at room temperature. As controls, mice were immunized with 10 μg freshly-prepared nanoparticles or 10 μg soluble 3HA protein. Serum samples were collected 2 weeks after priming and boosting immunizations and were assessed for anti-3HA IgG titers. No significant differences in IgG titer were observed between the nanoparticles stored at room temperature and freshly made nanoparticles. The double dose of aged nanoparticles induced significantly higher titers compared to soluble protein after the priming immunization, but that significance disappeared after the boost immunization (Fig 2).

**Fig 3:**
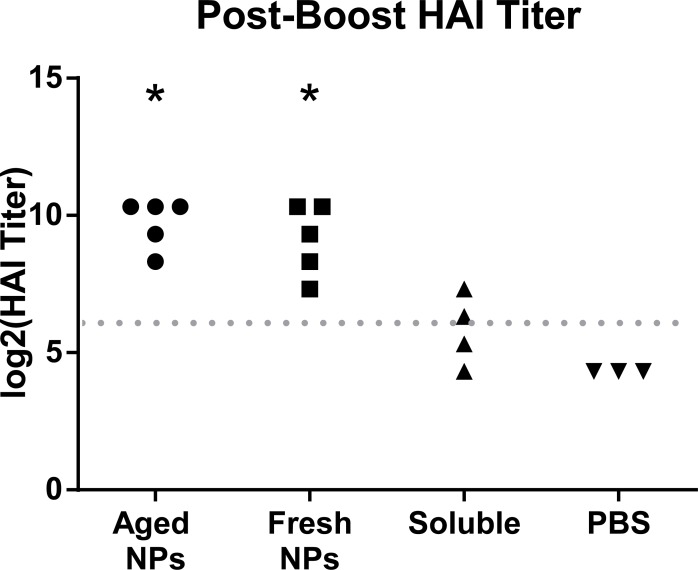
Aged and fresh nanoparticles induce hemagglutinin-inhibiting responses. Log of hemagglutination inhibition titer from mice post-boost immunization. Dotted line represents an HAI titer of 40, defined by the FDA to provide protective antibody responses[14]. * represents significantly different average titers from a titer of 40. Each data point represents a serum titer from one mouse. Limit of detection = 1:20 dilution.

Hemagglutination inhibition (HAI) titer is a measurement of the ability of serum antibodies to block hemagglutinin binding to sialic acid residues. This measurement is directly correlated to the ability of an animal’s antibodies to block influenza viruses from infecting host cells, and is a means of indirectly predicting neutralizing antibody production. The World Health Organization has defined a serum HAI titer of 40 or above to provide good protection against influenza infection[9]. Groups immunized with 10 μg stored and fresh nanoparticles had significantly higher HAI titers than 40, while groups immunized with soluble protein or PBS did not have significantly higher titers than 40 (Fig 3).

## Discussion

Nanoparticles were stored at room temperature for 112 days in PBS with no loss in hemagglutinating activity. According to a report by the United Nations Children’s Fund (UNICEF) and the WHO, approximately one month is needed for cold-chain transport of vaccines from a manufacturer in the developed world to a developing region[10]. Storage of protein nanoparticles for almost 3 times this length without additional excipients is especially promising, given that viral and subunit vaccine storage typically requires the addition of polyethylene glycol or sucrose to achieve weeks-long cold chain-independent storage[11, 12]. Surprisingly, soluble 3HA was also able to retain hemagglutinating activity for 56 days. This could be due to the inherent stability of this particular protein, and of recombinant subunit antigens generally[2].

Nanoparticles stored at 37°C in PBS were able to retain hemagglutinating activity for 2 weeks, but started to lose activity at 1 month. Although we did not immunize mice with nanoparticles stored at 37°C, we hypothesize that a similarly high antibody titer would be achieved based on the correlation between *in vitro* vaccine hemagglutinating activity and *in vivo* immunization efficacy[13]. Two weeks is a sufficient amount of time to reach rural health clinics from a distribution center[10], but may not be enough time to store the vaccine there for a prolonged period of time. Future studies should investigate the mechanism of elevated-temperature-related activity loss and whether stabilizing additives could mitigate any potential losses in immunogenicity.

Hemagglutination inhibition data suggested that our room temperature-stored nanoparticles could be just as effective at inducing neutralizing antibodies as freshly made nanoparticles. The FDA benchmark HAI titer of 40 allows us to compare our vaccine to an accepted standard of immunity[14], and our data suggests that our nanoparticles stored at room temperature can confer protective immunity. Given the ability of freshly-made 3HA nanoparticles to protect mice against a 10xLD_50_ challenge of H7N9 influenza[6], we relied on antibody measurements in this work. However, future challenge studies should examine whether nanoparticles stored at warm temperatures can also confer protective immunity.

The ability of 3HA nanoparticles to induce better HAI titers than soluble 3HA concurs with current nanoparticle vaccine literature theorizing that multivalent epitope presentation is a mechanism of recombinant antigen nanovaccine adjuvancy [2, 15, 16]. Our previous results demonstrating enhanced dendritic cell TNF-α and IL-1β responses to OVA-coated OVA protein nanoparticles also support this hypothesis[4].

In conclusion, we have shown that 3HA nanoparticles can be stored for up to 112 days at room temperature with no loss in *in vitro* hemagglutinating activity or *in vivo* antibody and hemagglutination-inhibiting responses. The fact that 3HA nanoparticles can remain immunogenic outside of cold-chain storage is a promising sign that nanoparticle vaccines made from recombinant protein antigens can survive both transport to and storage in the developing world. Future work in assessing and engineering protein nanoparticle vaccine stability should aim to (1) corroborate these results with other types of antigen nanoparticles, (2) examine the mechanism of elevated temperature-related antigen nanoparticle instability, and (3) extend viable storage at elevated temperatures beyond 2 weeks.

## Supporting information

S1 Fig Triplicate hemagglutination titer measurements of serial dilutions downwards of 3HA nanoparticles in PBS (Unlyo), water following lyophilization in an ammonium acetate buffer (AA-lyo), or water following lyophilization in PBS (PBS-lyo). (PDF)
