# Supplementary figures and images for: H7 Hemagglutinin nanoparticles retain immunogenicity after >3 months of 25°C storage
Source: PLoS One. 2018 Aug 9;13(8):e0202300. doi: 10.1371/journal.pone.0202300 (PMC6084952; doi:10.1371/journal.pone.0202300)

Buffer 1

Buffer 2

Buffer 3

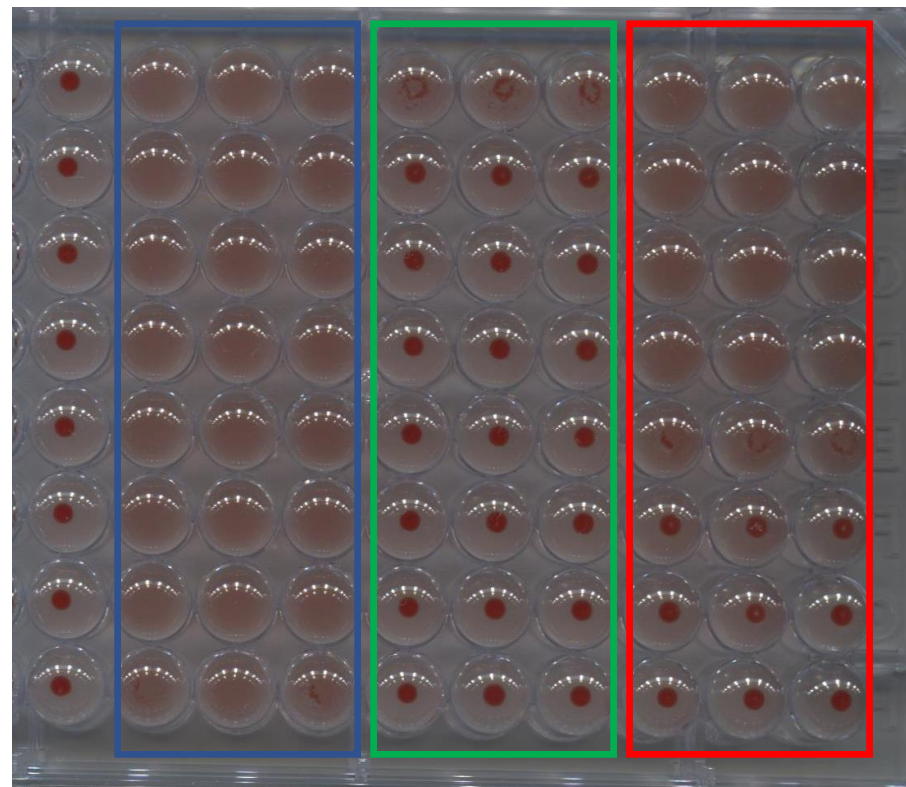

Supplement: S1 Fig — (PDF) [file pone.0202300.s001.pdf]
